# Analytical performances of Exacto^®^ HIV self-test in the Central African Republic

**DOI:** 10.11604/pamj.2022.41.236.31220

**Published:** 2022-03-22

**Authors:** Christian Diamant Mossoro-Kpinde, Christelle Bobossi, Serge Tonen-Wolyec, Ginette Claude Mireille Kalla, Coretha Baguida-Bokia, Simplice Sombot-Ndicki, Gerard Gresenguet, Francois-Xavier Mbopi-Keou, Laurent Belec

**Affiliations:** 1 Laboratoire National de Biologie Clinique et de Santé Publique, Bangui, République Centrafricaine,; 2 Faculté des Sciences de la Santé, Université de Bangui, Bangui, République Centrafricaine,; 3 Faculté de Médecine et de Pharmacie, Université de Kisangani, Kisangani, République Démocratique du Congo,; 4 Faculty of Medicine and Biomedical Sciences, University of Yaounde I, Yaounde, Cameroon,; 5 Laboratoire de Virologie, Hôpital Européen Georges Pompidou, Assistance Publique-Hôpitaux de Paris (AP-HP), Université de Paris, Paris Sorbonne Cité, Paris, France

**Keywords:** HIV, self-testing, rapid diagnostic test, immunochromatography, Central African Republic

## Abstract

We herein evaluated the analytical performances of the CE-IVD capillary blood Exacto^®^ HIV self-test (Biosynex, Strasbourg, France) in the Central African Republic (CAR). A cross-sectional study was conducted on a representative national panel of 200 sera positive for HIV and 200 negative for HIV, randomly selected thorough the CAR for HIV seroprevalence surveillance survey, according to reference test. The Exacto^®^ HIV self-test showed 99.5% (95% CI: 98.2-99.9) sensitivity and 100.0% (95% CI: 99.0-100.0) specificity. The Youden´s J index and Cohen´s Kappa coefficient were 0.995. At HIV-1 seroprevalence of 3.5% in the general adult population of the CAR, the positive and negative predictive values were 100% (95% CI: 99.0-100) and 99.9% (95% CI: 98.9-100), respectively. The results are within the limits required by the WHO (i.e. sensitivity ≥ 99.0% and specificity ≥ 98.0%), making Exacto^®^ HIV self-test suitable for routine use in the CAR.

## Introduction

The Central African Republic (CAR) has an adult HIV prevalence rate of 3.5% according UNAIDS´ updated data, with approximately 130,000 people living with HIV-1 [1]. Of these, approximately two-third are unaware of their HIV status [1]. Opportunities for HIV testing could be enhanced by offering accepted and effective strategies such as HIV self-testing in populations that fear stigma and discrimination when accessing conventional HIV counselling and testing in health care facilities [2, 3].

The CAR is continually carrying out new technologies for HIV testing in order to support the national HIV/AIDS policy. A recent study among lay users living in Bangui, the capital city of CAR, previously reported a high rate of the acceptability and the usability of the capillary blood-based Exacto^®^ HIV self-test (Biosynex, Strasbourg, France) [4]. Indeed, the Exacto ^®^HIV self-test is a third-generation immunochromatographic test, using a combination of a specific antibody binding protein that is conjugated to colloidal gold dye particles and synthetic antigens (gp41, gp36) able to detect antibodies (IgG and IgM) against HIV-1 or HIV-2 in whole-blood, serum or plasma, which are bound to the solid-phase membrane [3]. We herein investigated the analytical performances of the Exacto^®^ HIV self-test in the CAR, as recommended by the World Health Organization (WHO) before use [5].

## Methods

**Study design and setting:** this diagnostic performance evaluation was a cross-sectional study including plasma samples collected during the annual HIV seroprevalence surveillance survey from consenting adults from the general population from 2019. Eight field sites in Bangui *(Centre National de Références des Maladies Sexuellement Transmissibles et du SIDA, Complexe Pédiatrique, Hôpital de l´Amitié, Hôpital Communautaire, Centre de Santé de Castor, Centre de Santé de Lakouanga, Centre de Santé de Combatant, Centre de Santé de Ngaragba)*, the capital city of the CAR and seven health care centers (Berberati, Bimbo, Bossangoa, Bozoum, Bria, Damara, Mbaïki) of the main provincial cities of the country were drawn by the Ministry of Health for HIV seroprevalence surveillance survey.

**Sampling:** according to Centers for Disease Control and Prevention (CDC) and WHO recommendations, the minimum sample size for the study was estimated to be 200 HIV-positive and 200 HIV-negative plasma samples [6]. After listing all plasma samples that were previously positive or negative with the reference test, random sampling was used to select the 200 positive and 200 negative plasma samples to be tested with the Exacto^®^ HIV self-test.

**Laboratory procedures:** laboratory evaluation of the Exacto^®^ HIV self-test was carried out at the *Laboratoire National de Biologie Clinique et de Santé Publique* of Bangui. Indeed, a reference panel of plasma samples (200 positive and 200 negative) which had been tested according to reference national serological algorithm for HIV testing, using in parallel Genscreen ULTRA HIV Ag-Ab HIV-1/2 Version 2 (Bio-Rad, Marnes-la-Coquette, France) and Murex HIV 1.2.0 Ag/Ab Combination (Diasorin, Saluggia, Italy) was further tested with the Exacto^®^ HIV self-test. Exacto^®^ HIV self-test was performed using 5 μL of plasma without buffer, following the instructions of the manufacturer. The results of the study test were read by two clinical microbiologists blinded regarding the reference test results. Indeterminate readings were further read by a third microbiologist. Inconclusive plasmas were further tested by real time RT-PCR (Roche Molecular Systems, Inc., Branchburg, NJ, USA) for final diagnosis.

**Ethical considerations:** the study was approved by the Ethical and Scientific Committee, Faculty of Health Sciences of Bangui, constituting the Institutional Ethical Committee.

**Statistical analysis:** data were entered into an Excel database (Redmond, WA, USA) and analysed using SPSS 20.0 (Chicago, IL, USA). Analytical performances of Exacto^®^ HIV self-test was defined using sensitivity, specificity, concordance, accuracy, positive predictive values (PPV) and negative predictive values (NPV) as previously described [7]. Briefly, the Cohen´s Kappa coefficient calculation was used to estimate the concordance, and interpreted according to the Landis and Koch scale; accuracy was estimated by Youden´s J index; the PPV and NPV were calculated by using Bayes´ formulae taking into account of the HIV prevalence of 3.5% in the CAR.

## Results

The results of the analytical performances of Exacto^®^ HIV self-test are depicted in Table 1. Among the 200 HIV samples known to be HIV-positive with reference test, only 1 was negative by Exacto^®^ HIV self-test, but positive by RT-PCR, and was thus considered as false negative result. Thus, a total of 199 plasma samples of 200 true-positive plasma samples were found positive by Exacto^®^ HIV self-test, while 200 plasmas of the 200 true-negative plasmas samples were all found negative with Exacto^®^ HIV self-test. Taken together, the sensitivity and specificity of the Exacto^®^ HIV self-test were 99.5% (95% CI: 98.2-99.9), and 100% (95%CI: 99.0-100), respectively.

**Table 1:** analytical performances of Exacto^®^ HIV self-test

	Reference algorithm for HIV testing		
	Positive Number	Negative Number	Total Number
**Exacto**^®^ **HIV self-test results**			
Positive	199	0	199
Negative	1	200	201
Total	200	200	400
**Performances**, *% (95% CI) ^#^*			
Sensitivity	99.5 (98.2 – 99.9)		
Specificity	100 (99.0 – 100)		
Cohen's k coefficient^$^	0.95 (0.98 – 1.0)		
Youden’s J index^£^	99.5 (98.2–99.9)		
PPV^β^	100 (99.0–100)		
NPV^β^	99.9 (98.9–100)		

# The results were presented as a 95% confidence interval (CI) using the Wilson score bounds; ^$^The Cohen's K coefficient calculation was used to estimate the concordance, and interpreted according the Landis and Koch scale as follows: < 0 as indicating no agreement, 0–0.20 as slight, 0.21–0.40 as fair, 0.41–0.60 as moderate, 0.61–0.80 as substantial, and 0.81–1 as almost perfect agreement; ^£^ Accuracy was estimated by Youden's J index = sensitivity + specificity – 1; accuracy is expressed in percentage; ^β^ The positive predictive values (PPV) and negative predictive values (NPV) were calculated by using Bayes' formulae taking account of the HIV prevalence of 3.5% in the Central Africa Republic

The reliability of Exacto^®^ HIV self-test estimated to 99.5% (95% CI: 98.2-99.9) by the Cohen´s Kappa coefficient measuring the concordance between the HIV self-test and reference serological algorithm, demonstrating almost perfect agreement according to Landis and Koch scale. The accuracy of Exacto^®^ HIV self-test estimated to 99.5% (95% CI: 98.2-99.9) by the Youden's J index, demonstrating excellent accuracy. At HIV-1 seroprevalence of 3.5% in general adult population of the CAR in 2019, the positive and negative predictive values were 100% (95% CI: 99.0-100) and 99.98% (95% CI: 98.9-100), respectively.

## Discussion

We herein evaluated the analytical performances of the Exacto^®^ HIV self-test (Biosynex, Strasbourg, France) in the CAR on a representative national panel. The Exacto^®^ HIV self-test showed high sensitivity (99.5%), specificity (100%), concordance, accuracy and positive and negative predictive values.

The virological performances of HIV self-test in French-speaking countries in sub-Saharan Africa are yet poorly established. In practice, clinical validation of the analytical performances of HIV self-test in real-life should be always carried out in a sufficiently large number of target population subjects before introducing them into the routine diagnosis, as strongly recommended by the WHO [8].

In addition, Central Africa is characterized by broad genetic diversity of HIV-1 strains, which can be associated with false negativity of HIV immunochromatographic rapid diagnostic tests [9], and by a variety of factors which can be associated with false positivity or unspecific reactivities, including disturbances affecting the B cell-driven immunity during infectious diseases, such as marked immunological stimulation, strong non-specific polyclonal B-cell activation, hypergammaglobulinemia, and production of circulating immune complexes [10].

HIV self-tests are more and more developed and may be of variable quality [7]. In the present study, the analytical performances of Exacto^®^ HIV self-test in the CAR, were evaluated using a collection of 200 positive and 200 negative sera randomly selected thorough the CAR for HIV seroprevalence surveillance survey. The results showed excellent analytical performances of the Exacto^®^ HIV self-test, despite the risk of false-positive results with frequent inconclusive sera in this area of Africa [5, 10]. Only one positive for HIV was found negative by the HIV self-test, but the final estimated sensitivity remained high. Finally, the analytical performances of Exacto^®^ HIV self-test were within the limits required by the WHO for HIV self-testing i.e. sensitivity ≥ 99.0% and specificity ≥ 98.0% [8], likely allowing it to detect HIV-1 strains circulating in the CAR. A previous study conducted in the Democratic Republic of the Congo showed similar high analytical performances of the Exacto^®^ HIV self-test [3].

This study has some limitations. This study did not consider the possibility of misinterpretation by lay users, as previously described. Indeed, under field conditions, misinterpretation of the HIV self-test could alter the proportion of falsely interpreted positive and negative results, thus reducing its analytical performance in the hands of lay users.

## Conclusion

In conclusion, the Exacto^®^ HIV self-test showed high sensitivity and specificity, making the test within the analytical limits required by the WHO to be used in routine in the general population for HIV testing in CAR. Taken together, the high analytical performances in addition to the previously reported high practicability and usability of the Exacto^®^ HIV self-test make the test suitable for routine use in the CAR.

### What is known about this topic

- *Field validation of capillary HIV Self-Test constitutes a mandatory prerequisite before use;*
- *Broad spectrum of HIV-1 strains are circulating in Central Africa;*
- *HIV rapid test may lack specificity in the Central African context*.

### What this study adds

- *Exacto® HIV Self-Test is highly sensitive and specific in Central Africa;*
- *Exacto® HIV Self-Test can be used safely by adult profane public with high confidence in Central Africa*.
